# Effect of MgCl_2_ and GdCl_3_ on ORAI1 Expression and Store-Operated Ca^2+^ Entry in Megakaryocytes

**DOI:** 10.3390/ijms22073292

**Published:** 2021-03-24

**Authors:** Kuo Zhou, Xuexue Zhu, Ke Ma, Jibin Liu, Bernd Nürnberg, Meinrad Gawaz, Florian Lang

**Affiliations:** 1 Department of Pharmacology, Experimental Therapy & Toxicology, Eberhard Karls University, 72074 Tübingen, Germany; azh.zhoukuo@gmail.com (K.Z.); xuexue.zhu1992@gmail.com (X.Z.); mke_card@163.com (K.M.); bernd.nuernberg@uni-tuebingen.de (B.N.); 2 Institute of Preventive Veterinary Medicine, Key Laboratory of Animal Disease and Human Health of Sichuan Province, Sichuan Agricultural University, Chengdu 611130, China; eaeas12@163.com; 3 Department of Cardiology and Angiology, University Hospital Tübingen, Eberhard Karls University Tübingen, 72076 Tübingen, Germany; meinrad.gawaz@med.uni-tuebingen.de; 4 Department of Vegetative and Clinical Physiology, Eberhard Karls University, 72074 Tübingen, Germany

**Keywords:** SOCE, ORAI1,2,3, STIM1,2, SGK1, NFAT5, Mg^2+^, Gd^3+^, calcium-sensing receptor, megakaryocytes

## Abstract

In chronic kidney disease, hyperphosphatemia upregulates the Ca^2+^ channel ORAI and its activating Ca^2+^ sensor STIM in megakaryocytes and platelets. ORAI1 and STIM1 accomplish store-operated Ca^2+^ entry (SOCE) and play a key role in platelet activation. Signaling linking phosphate to upregulation of ORAI1 and STIM1 includes transcription factor NFAT5 and serum and glucocorticoid-inducible kinase SGK1. In vascular smooth muscle cells, the effect of hyperphosphatemia on ORAI1/STIM1 expression and SOCE is suppressed by Mg^2+^ and the calcium-sensing receptor (CaSR) agonist Gd^3+^. The present study explored whether sustained exposure to Mg^2+^ or Gd^3+^ interferes with the phosphate-induced upregulation of NFAT5, SGK1, ORAI1,2,3, STIM1,2 and SOCE in megakaryocytes. To this end, human megakaryocytic Meg-01 cells were treated with 2 mM ß-glycerophosphate for 24 h in the absence and presence of either 1.5 mM MgCl_2_ or 50 µM GdCl_3_. Transcript levels were estimated utilizing q-RT-PCR, protein abundance by Western blotting, cytosolic Ca^2+^ concentration ([Ca^2+^]_i_) by Fura-2 fluorescence and SOCE from the increase in [Ca^2+^]_i_ following re-addition of extracellular Ca^2+^ after store depletion with thapsigargin (1 µM). As a result, Mg^2+^ and Gd^3+^ upregulated CaSR and blunted or virtually abolished the phosphate-induced upregulation of NFAT5, SGK1, ORAI1,2,3, STIM1,2 and SOCE in megakaryocytes. In conclusion, Mg^2+^ and the CaSR agonist Gd^3+^ interfere with phosphate-induced dysregulation of [Ca^2+^]_i_ in megakaryocytes.

## 1. Introduction

The impairment of renal phosphate excretion in chronic kidney disease (CKD) increases the plasma phosphate concentration with subsequent osteogenic reprogramming of vascular smooth muscle cells (VSMCs) [1,2,3,4] and vascular calcification [5,6,7,8]. Underlying signaling includes nuclear factor of activated T cells 5 (NFAT5) [9,10,11,12], serum and glucocorticoid-inducible kinase 1 (SGK1) [13,14] and Ca^2+^ channel ORAI with its activator stromal interaction molecule (STIM) [15,16,17]. Opening of ORAI by STIM upon intracellular Ca^2+^ depletion results in store-operated Ca^2+^ entry (SOCE) [15], which contributes to the orchestration of vascular calcification [16,17,18] as well as mineralization of bone [19] and enamel [20,21].

NFAT5, SGK1 and ORAI1/STIM1 similarly participate in activation of blood platelets [22,23,24,25,26,27] and thus contribute to the development of thrombosis and thrombo-occlusive events [23,26,27]. Platelets are generated by megakaryocytes, and protein abundance in platelets depends on megakaryocytic transcript and protein expression [28,29]. Megakaryocytes express ORAI1, ORAI2 and ORAI3 [30], thus accomplishing SOCE [31,32,33,34]. ORAI expression and SOCE are upregulated by the phosphate donor ß-glycerophosphate [30]. The stimulation of vascular calcification by phosphate has been shown to be reversed by Mg^2+^ and the calcium-sensing receptor (CaSR) agonist Gd^3+^ [17,35].

Expression of the calcium-sensing receptor has previously been shown in both megakaryocytes and platelets [36]. CaSR activation has been shown to counteract activation of blood platelets in hyperhomocysteinemia [37]. However, to the best of our knowledge, a functional role of CaSR in megakaryocytes and a role of CaSR in the regulation of ORAI1/STIM1 abundance and activity of megakaryocytes and platelets have never been reported before. The present paper thus explored whether Mg^2+^ or Gd^3+^ modifies NFAT5, SGK1, ORAI1,2,3 and STIM1,2 expression as well as SOCE in megakaryocytes without or with prior exposure to phosphate donor ß-glycerophosphate.

## 2. Results

**Figure 1:**
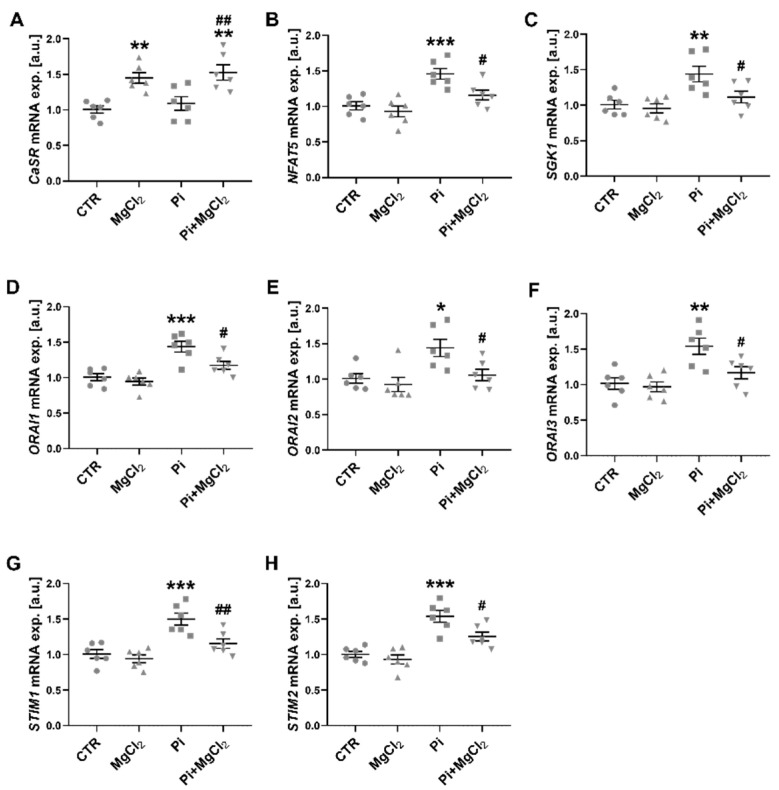
Upregulation of *CaSR* transcription by MgCl_2_ and reversal of ß-glycerophosphate-induced *NFAT5*, *SGK1*, *ORAI1*, *ORAI2*, *ORAI3*, *STIM1* and *STIM2* transcription in megakaryocytes by MgCl_2_. (**A**–**H**) Arithmetic means (±SEM, *n* = 6) of *CaSR* (**A**), *NFAT5* (**B**), *SGK1* (**C**), *ORAI1* (**D**), *ORAI2* (**E**), *ORAI3* (**F**), *STIM1* (**G**) and *STIM2* (**H**) transcript levels in megakaryocytes without (CTR) and with prior 24-h exposure to 1.5 mM MgCl_2_ alone (MgCl_2_), 2 mM ß-glycerophosphate alone (Pi) or 2 mM ß-glycerophosphate and 1.5 mM MgCl_2_ (Pi+MgCl_2_). * (*p* < 0.05), ** (*p* < 0.01), *** (*p* < 0.001) indicate statistically significant differences compared to CTR; # (*p* < 0.05), ## (*p* < 0.01) indicate statistically significant differences compared to Pi alone (ANOVA). CaSR, calcium-sensing receptor; CTR, control.

In the first set of experiments, RT-PCR was employed to quantify transcript levels encoding the calcium-sensing receptor (*CaSR*), the transcription factor *NFAT5*, the NFAT5-regulated *SGK1*, the SGK1-sensitive Ca^2+^ release-activated ion channels *ORAI1*, *ORAI2* and *ORAI3* and the ORAI-activating Ca^2+^ sensor isoforms *STIM1* and *STIM2*. According to RT-PCR (Figure S1), *ORAI1* was, by far, the predominant *ORAI* isoform and *STIM1* the prevailing *STIM* isoform. As shown in Figure 1, MgCl_2_ treatment significantly upregulated *CaSR* expression with or without prior exposure to ß-glycerophosphate. In agreement with earlier observations [30], a 24-h pretreatment of human megakaryocytes with the phosphate donor ß-glycerophosphate upregulated the transcript levels of *NFAT5*, *SGK1*, *ORAI1*, *ORAI2*, *ORAI3*, *STIM1* and *STIM2*. All those effects were significantly blunted or virtually abrogated by additional exposure to 1.5 mM MgCl_2_. In the absence of ß-glycerophosphate, MgCl_2_ did not significantly modify the transcript levels of *NFAT5*, *SGK1*, *ORAI1*, *ORAI2*, *ORAI3*, *STIM1* and *STIM2* (Figure 1).

**Figure 2:**
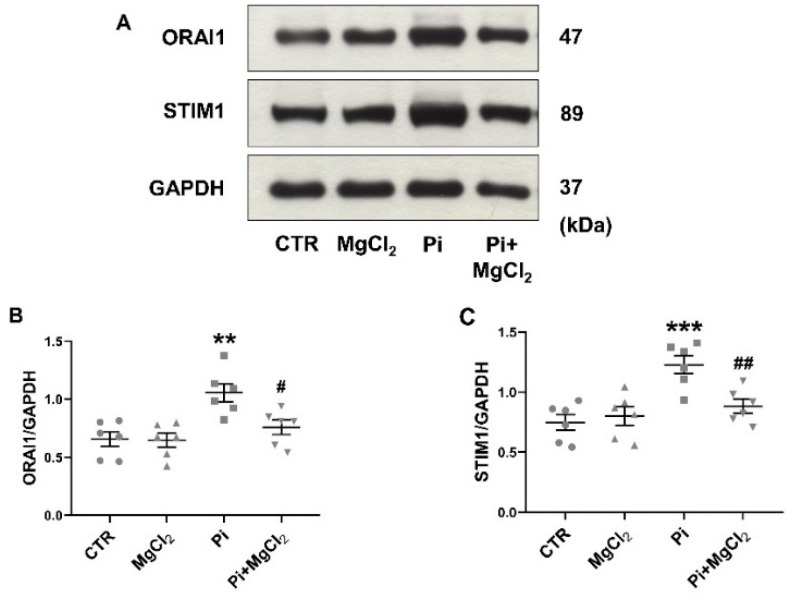
Reversal of ß-glycerophosphate-induced ORAI1 and STIM1 protein expression in megakaryocytes by MgCl_2_. (**A**) Original Western blots of ORAI1, STIM1 and GAPDH protein abundance in megakaryocytes without (CTR) and with prior 24-h exposure to 1.5 mM MgCl_2_ alone (MgCl_2_), 2 mM ß-glycerophosphate alone (Pi) or 2 mM ß-glycerophosphate and 1.5 mM MgCl_2_ (Pi+MgCl_2_). (**B**,**C**) Arithmetic means ± SEM (*n* = 6) of ORAI1 (**B**) and STIM1 (**C**) protein abundance in megakaryocytes without (CTR) and with prior 24-h exposure to 1.5 mM MgCl_2_ alone (MgCl_2_), 2 mM ß-glycerophosphate alone (Pi) or 2 mM ß-glycerophosphate and 1.5 mM MgCl_2_ (Pi+MgCl_2_). ** (*p* < 0.01), *** (*p* < 0.001) indicate statistically significant differences compared to CTR; # (*p* < 0.05), ## (*p* < 0.01) indicate statistically significant differences compared to Pi alone (ANOVA). CTR, control.

Western blots were performed to test whether the observed alterations in ORAI1 and STIM1 transcript levels are paralleled by the respective changes in ORAI1 and STIM1 protein abundance. As illustrated in Figure 2, ß-glycerophosphate upregulated the ORAI1 and STIM1 protein abundance, an effect significantly blunted by additional exposure to 1.5 mM MgCl_2_. Again, in the absence of ß-glycerophosphate, MgCl_2_ did not significantly modify the ORAI1 and STIM1 protein abundance (Figure 2).

**Figure 3:**
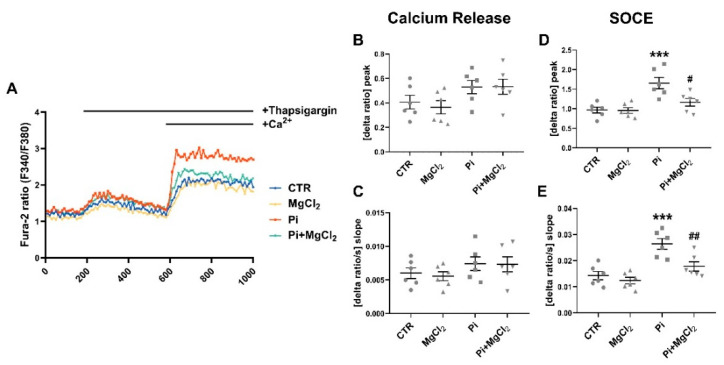
Reversal of ß-glycerophosphate-induced increase in store-operated Ca^2+^ entry (SOCE) in megakaryocytes by MgCl_2_. (**A**) Representative tracings of Fura-2 fluorescence ratio in fluorescence spectrometry before and following extracellular Ca^2+^ removal and addition of thapsigargin (1 µM) as well as re-addition of extracellular Ca^2+^ in megakaryocytes without (CTR) or with prior 24-h exposure to 1.5 mM MgCl_2_ alone (MgCl_2_), 2 mM ß-glycerophosphate alone (Pi) or 2 mM ß-glycerophosphate and 1.5 mM MgCl_2_ (Pi+MgCl_2_). (**B**,**C**) Arithmetic means (±SEM, *n* = 6) of peak (**B**) and slope (**C**) increase in Fura-2 fluorescence ratio following addition of thapsigargin (1 µM) in megakaryocytes without (CTR) or with prior 24-h exposure to 1.5 mM MgCl_2_ alone (MgCl_2_), 2 mM ß-glycerophosphate alone (Pi) or 2 mM ß-glycerophosphate and 1.5 mM MgCl_2_ (Pi+MgCl_2_). (**D**,**E**) Arithmetic means (±SEM, *n* = 6) of peak (**D**) and slope (**E**) increase in Fura-2 fluorescence ratio following re-addition of extracellular Ca^2+^ in megakaryocytes without (CTR) or with prior 24-h exposure to 1.5 mM MgCl_2_ alone (MgCl_2_), 2 mM ß-glycerophosphate alone (Pi) or 2 mM ß-glycerophosphate and 1.5 mM MgCl_2_ (Pi+MgCl_2_). *** (*p* < 0.001) indicates statistically significant difference compared to CTR; # (*p* < 0.05), ## (*p* < 0.01) indicate statistically significant differences compared to Pi alone (ANOVA). CTR, control.

Cytosolic Ca^2+^ activity ([Ca^2+^]_i_) was estimated utilizing Fura-2 fluorescence to test whether the enhanced expression of ORAI and STIM is followed by the respective alterations in store-operated Ca^2+^ entry (SOCE). The 340 nm/380 nm ratio reflecting [Ca^2+^]_i_ was, prior to store depletion, similar in ß-glycerophosphate-treated (1.298 ± 0.086, *n* = 6) and untreated (1.202 ± 0.094, *n* = 6) megakaryocytes. For determination of SOCE, cells were exposed to thapsigargin (1 µM), a sarco/endoplasmic reticulum Ca^2+^/ATPase (SERCA) inhibitor, and Ca^2+^-free solutions to deplete intracellular Ca^2+^ stores. In the following, extracellular Ca^2+^ was re-added in the continued presence of thapsigargin to quantify SOCE from the increase in [Ca^2+^]_i_. As illustrated in Figure 3, ß-glycerophosphate pretreatment significantly increased both the peak and the slope of SOCE, an effect significantly blunted by additional exposure to 1.5 mM MgCl_2_. Again, in the absence of ß-glycerophosphate, MgCl_2_ did not significantly modify SOCE. Neither ß-glycerophosphate nor MgCl_2_ significantly modified Ca^2+^ release.

**Figure 4:**
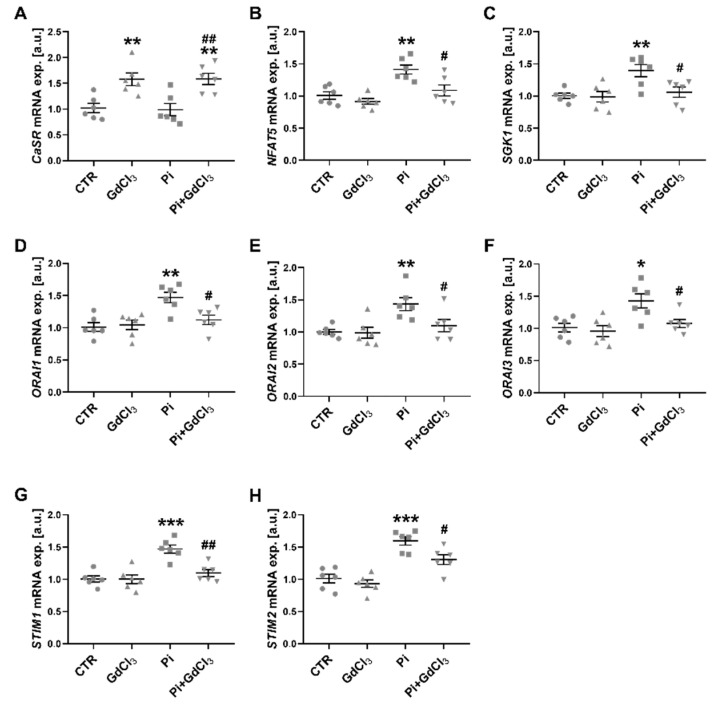
Upregulation of *CaSR* transcription by GdCl_3_ and reversal of ß-glycerophosphate-induced *NFAT5*, *SGK1*, *ORAI1*, *ORAI2*, *ORAI3*, *STIM1* and *STIM2* transcription in megakaryocytes by GdCl_3_. (A–H) Arithmetic means (±SEM, *n* = 6) of *CaSR* (**A**), *NFAT5* (**B**), *SGK1* (**C**), *ORAI1* (**D**), *ORAI2* (**E**), *ORAI3* (**F**), *STIM1* (**G**) and *STIM2* (**H**) transcript levels in megakaryocytes without (CTR) and with prior 24-h exposure to 50 µM GdCl_3_ alone (GdCl_3_), 2 mM ß-glycerophosphate alone (Pi) or 2 mM ß-glycerophosphate and 50 µM GdCl_3_ (Pi+GdCl_3_). * (*p* < 0.05), ** (*p* < 0.01), *** (*p* < 0.001) indicate statistically significant differences compared to CTR; # (*p* < 0.05), ## (*p* < 0.01) indicate statistically significant differences compared to Pi alone (ANOVA). CaSR, calcium-sensing receptor; CTR, control.

Additional experiments were performed to test whether the effects of MgCl_2_ were mimicked by calcium-sensing receptor agonist Gd^3+^. As illustrated in Figure 4, *CaSR* transcription was significantly upregulated by the exposure to 50 µM GdCl_3_ both in the absence and presence of ß-glycerophosphate. The upregulation of *NFAT5*, *SGK1*, *ORAI1*, *ORAI2*, *ORAI3*, *STIM1* and *STIM2* transcript levels by 2 mM ß-glycerophosphate was significantly blunted by additional exposure to 50 µM GdCl_3_. In the absence of ß-glycerophosphate, 50 µM GdCl_3_ did not significantly modify the transcript levels of *NFAT5*, *SGK1*, *ORAI1*, *ORAI2*, *ORAI3*, *STIM1* and *STIM2* (Figure 4).

**Figure 5:**
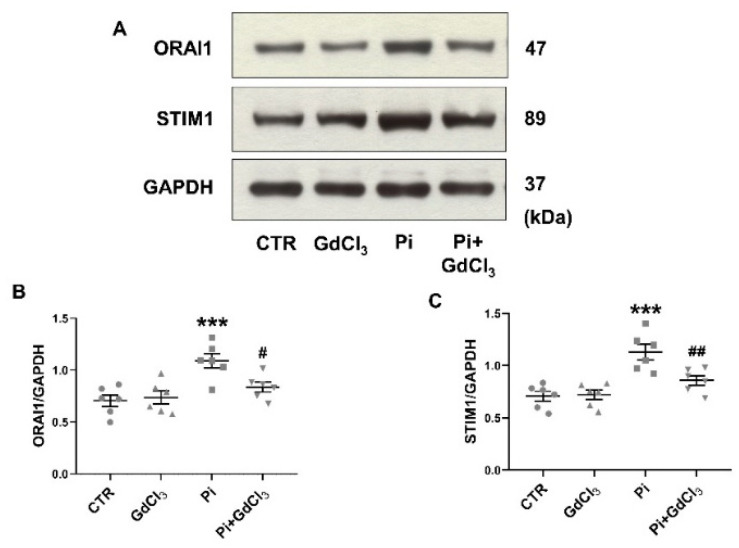
Reversal of ß-glycerophosphate-induced ORAI1 and STIM1 protein expression in megakaryocytes by GdCl_3_. (**A**) Original Western blots of ORAI1, STIM1 and GAPDH protein abundance in megakaryocytes without (CTR) and with prior 24-h exposure to 50 µM GdCl_3_ alone (GdCl_3_), 2 mM ß-glycerophosphate alone (Pi) or 2 mM ß-glycerophosphate and 50 µM GdCl_3_ (Pi+GdCl_3_). (**B**,**C**) Arithmetic means ± SEM (*n* = 6) of ORAI1 (**B**) and STIM1 (**C**) protein abundance in megakaryocytes without (CTR) and with prior 24-h exposure to 50 µM GdCl_3_ alone (GdCl_3_), 2 mM ß-glycerophosphate alone (Pi) or 2 mM ß-glycerophosphate and 50 µM GdCl_3_ (Pi+GdCl_3_). *** (*p* < 0.001) indicates statistically significant difference compared to CTR; # (*p* < 0.05), ## (*p* < 0.01) indicate statistically significant differences compared to Pi alone (ANOVA). CTR, control.

As shown in Figure 5, 50 µM GdCl_3_ further significantly blunted the ß-glycerophosphate-induced upregulation of ORAI1 and STIM1 protein abundance (Figure 5). Again, 50 µM GdCl_3_ did not significantly modify the ORAI1 and STIM1 protein abundance in the absence of ß-glycerophosphate (Figure 5).

**Figure 6:**
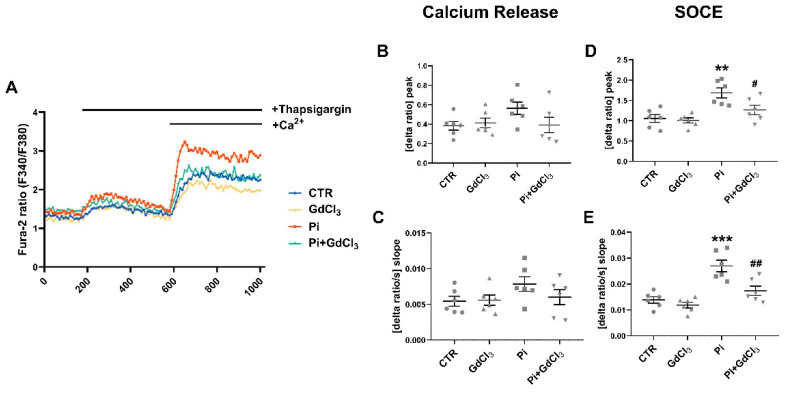
Reversal of ß-glycerophosphate-induced increase in store-operated Ca^2+^ entry (SOCE) in megakaryocytes by GdCl_3_. (**A**) Representative tracings of Fura-2 fluorescence ratio in fluorescence spectrometry before and following extracellular Ca^2+^ removal and addition of thapsigargin (1 µM) as well as re-addition of extracellular Ca^2+^ in megakaryocytes without (CTR) or with prior 24-h exposure to 50 µM GdCl_3_ alone (GdCl_3_), 2 mM ß-glycerophosphate alone (Pi) or 2 mM ß-glycerophosphate and 50 µM GdCl_3_ (Pi+GdCl_3_). (**B**,**C**) Arithmetic means (±SEM, *n* = 6) of peak (**B**) and slope (**C**) increase in Fura-2 fluorescence ratio following addition of thapsigargin (1 µM) in megakaryocytes without (CTR) or with prior 24-h exposure to 50 µM GdCl_3_ alone (GdCl_3_), 2 mM ß-glycerophosphate alone (Pi) or 2 mM ß-glycerophosphate and 50 µM GdCl_3_ (Pi+GdCl_3_). (**D**,**E**) Arithmetic means (±SEM, *n* = 6) of peak (**D**) and slope (**E**) increase in Fura-2 fluorescence ratio following re-addition of extracellular Ca^2+^ in megakaryocytes without (CTR) or with prior 24-h exposure to 50 µM mM GdCl_3_ alone (GdCl_3_), 2 mM ß-glycerophosphate alone (Pi) or 2 mM ß-glycerophosphate and 50 µM GdCl_3_ (Pi+GdCl_3_). ** (*p* < 0.01), *** (*p* < 0.001) indicate statistically significant differences compared to CTR; # (*p* < 0.05), ## (*p* < 0.01) indicate statistically significant differences compared to Pi alone (ANOVA). CTR, control.

As illustrated in Figure 6, 50 µM GdCl_3_ significantly blunted the ß-glycerophosphate-induced upregulation of the SOCE peak and slope without significantly modifying Ca^2+^ release. Again, in the absence of ß-glycerophosphate, 50 µM GdCl_3_ did not significantly modify SOCE.

## 3. Discussion

The present study confirms previous observations [30] demonstrating the upregulation of NFAT5, ORAI1, ORAI2, ORAI3, STIM1 and STIM2 expression as well as SOCE by the phosphate donor ß-glycerophosphate in megakaryocytes.

More importantly, the present study reveals that all those effects were blunted or virtually abolished by 1.5 mM MgCl_2_ and by 50 µM GdCl_3_. MgCl_2_ and GdCl_3_ are, at least in part, effective by suppression of NFAT5 expression. NFAT5 upregulates the expression of SGK1 [13], which fosters degradation of the inhibitor protein IκBα and subsequent nuclear translocation of the transcription factor NFκB [15]. NFκB is a powerful stimulator of ORAI1 expression [15]. The present observations do not, however, exclude further signaling contributing to the upregulation of ORAI/STIM by ß-glycerophosphate and its reversal by MgCl_2_ or GdCl_3_ in megakaryocytes. It should be pointed out that the signaling shown here is triggered by sustained exposure to MgCl_2_ or GdCl_3_. Different signaling elements may prevail following acute stimulation with MgCl_2_ or GdCl_3_, such as G protein-dependent activation of phospholipase C and inositol trisphosphate formation, as shown in other cell types [38,39,40,41,42,43].

Without ß-glycerophosphate treatment, the addition of 50 µM GdCl3 did not significantly modify SOCE (Figure 6). The possibility should be considered that CaSR activation disrupts the upregulation of ORAI by ß-glycerophosphate/NFAT5/NFκB but does not modify basal ORAI expression. However, the scatter of the data does not allow exclusion of minor effects.

ORAI and STIM participate in the orchestration of platelet activation [44]. At least in theory, inhibition of ORAI and STIM expression and SOCE could contribute to the previously observed inhibition of platelet activity by CaSR activation in hyperhomocysteinemia [37]. Moreover, in view of the present observations and the known role of platelets in the pathophysiology of cardiac infarction and stroke [45], CaSR activation could counteract upregulation of platelet activity by phosphate and thus reduce the risk of cardiac infarction and stroke in CKD patients [46,47]. Clearly, additional experimental effort and clinical studies are required to define the potentially protective effect of CaSR agonists on pathological platelet activity in CKD.

**Figure 7:**
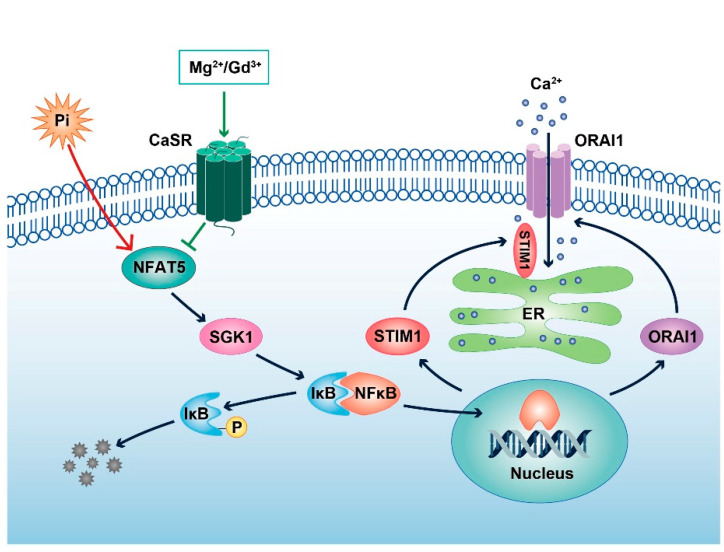
Mg^2+^ and Gd^3+^ interfere with phosphate-induced SOCE upregulation by the activation of CaSR in megakaryocytes—a schematic representation. In megakaryocytes, the phosphate donor ß-glycerophosphate upregulates NFAT5 and SGK1 expression, which leads to the degradation of the NFκB inhibitory protein IκB with subsequent nuclear translocation of NFκB and NFκB-dependent transcription of ORAI1 and STIM1. CaSR can be activated by cations such as Mg^2+^ and Gd^3+^ and inhibits phosphate-induced SOCE enhancement, at least partially, via the downregulation of the signaling pathway. CaSR, calcium-sensing receptor; ER, endoplasmic reticulum.

In conclusion, the activation of CaSR by MgCl_2_ and GdCl_3_ reverses the upregulation of NFAT5, SGK1, ORAI1, ORAI2, ORAI3, STIM1 and STIM2 expression as well as SOCE by the phosphate donor ß-glycerophosphate in megakaryocytes (Figure 7). The effect may decrease the cardiovascular risk in hyperphosphatemic chronic kidney disease patients.

## 4. Materials and Methods

### 4.1. Cell Culture

Human megakaryocytic cells (Meg-01) from American Type Culture Collection (ATCC, Manassas, VA, USA) were cultured in RPMI 1640 (Roswell Park Memorial Institute medium, Gibco, Paisley, UK) containing 10% fetal bovine serum (FBS, Gibco, Paisley, United Kingdom) and 1% Penicillin/Streptomycin in a humidified incubator at 37ºC and 5% CO_2_. Where indicated, the cells were exposed to 2 mM ß-glycerophosphate (Sigma, Steinheim, Germany) for 24 h in the absence and presence of 1.5 mM MgCl_2_ or 50 µM GdCl_3_ (Sigma, Steinheim, Germany). In analysis of vascular calcification, phosphate donor ß-glycerophosphate is widely used as a substitute for phosphate and a well-established stimulator of tissue calcification [48,49,50].

### 4.2. Quantitative PCR

To determine transcript levels of *CaSR*, *NFAT5*, *SGK1*, *ORAI1*, *ORAI2*, *ORAI3*, *STIM1* and *STIM2*, total RNA was extracted according to the manufacturer’s instructions with TriFast (Peqlab, Erlangen, Germany) [16,51,52,53,54]. DNAse digestion was performed to avoid DNA contamination and was followed by reverse transcription using Oligo(dT)15 primers (Promega, Mannheim, Germany) and the GoScript reverse transcription system (Promega, Mannheim, Germany). Real-time polymerase chain reaction (RT-PCR) amplification of the respective genes was set up in a total volume of 15 µL using 100 ng of cDNA, 500 nM forward and reverse primers and 2× GoTaq^®^ qPCR Master Mix (Promega, Hilden, Germany) following the manufacturer’s protocol. Cycling conditions were as follows: initial denaturation at 95 °C for 3 min, followed by 40 cycles of 95 °C for 15 s, 60 °C for 30 s and 72 °C for 30 s. The primers used for amplification in this study are given in Table 1 (Invitrogen, Darmstadt, Germany).

Melting curves were analyzed to confirm PCR product specificity. The CFX96 Real-Time System (BioRad, Munich, Germany) was used to perform real-time PCR amplifications. All experiments were conducted in duplicate. Relative mRNA expression was calculated by the 2^−ΔΔCT^ method using the housekeeping gene Glyceraldehyde 3-phosphate dehydrogenase (*GAPDH*) as internal reference, normalized to the control group.

### 4.3. Western Blotting

Protein abundance of ORAI1, STIM1 and GAPDH was determined by Western blotting [16,51,52,53,54]. Megakaryocyte suspensions were centrifuged for 5 min at 300× *g* and 4 °C. The pellet was washed with ice-cold PBS and suspended in 40 μL ice-cold RIPA lysis buffer (Cell Signaling Technology, Danvers, MA, USA) containing Protease Inhibitor Cocktail (Thermo-Fisher Scientific, Waltham, MA, USA). After centrifugation (20,000× *g*, 4 °C for 20 min), the supernatant was taken to determine protein concentration using the Bradford assay (BioRad, Munich, Germany). For Western blotting, 30 µg of proteins was electro-transferred onto a poly-vinylidene difluoride (PVDF) membrane after electrophoresis using 10% SDS-PAGE and blocked with 5% milk in TBST at room temperature for 1 h. The membranes were incubated with primary anti-ORAI1 antibody (1:1000, Proteintech, Chicago, IL, USA), anti-STIM1 antibody (1:1000, Cell Signaling Technology, Danvers, MA, USA) and anti-GAPDH antibody (1:2000, Cell Signaling Technology, Danvers, MA, USA) at 4 °C overnight. After washing (TBST), the blots were incubated with secondary anti-rabbit antibody conjugated with horseradish peroxidase (1:2000, Cell Signaling Technology, Danvers, MA, USA) for 2 h at room temperature. Protein bands were detected after additional washes (TBST) with an ECL detection reagent (Thermo-Fisher Scientific, Waltham, MA, USA). For densitometry image analysis, Western blots were scanned and analyzed by ImageJ software (Version 1.52, NIH, Bethesda, MD, USA). The results are shown as the ratio of total protein to GAPDH. Protein-Marker (Thermo-Fisher Scientific, Waltham, MA, USA) was used as reference to assign the right protein size.

### 4.4. Cytosolic Calcium Measurements

To determine the cytosolic Ca^2+^ concentration ([Ca^2+^]_i_), Fura-2 fluorescence was utilized [16,51,52,53,54,55]. Cells were preincubated for 30–45 min with Fura-2/AM (2 µM, Invitrogen, Goettingen, Germany) at 37 °C and excited alternatively at 340 nm and 380 nm in an inverted phase-contrast microscope (Axiovert 100, Zeiss, Oberkochen, Germany) through an objective (Fluor 40×/1.30 oil). At 505 nm, the emitted fluorescence intensity was recorded. Data (6/minute) were acquired using computer software Metafluor (Version 7.5, Universal Imaging, Downingtown, PA, USA). To estimate cytosolic Ca^2+^ activity, ratiometer (340 nm/380 nm)-based analysis was employed. SOCE was determined following extracellular Ca^2+^ removal causing store depletion and subsequent Ca^2+^ re-addition in constant presence of SERCA inhibitor thapsigargin (1 µM, Invitrogen, Goettingen, Germany). For quantification of Ca^2+^ entry, the slope (delta ratio/s) and peak (delta ratio) were determined following re-addition of Ca^2+^. Experiments were performed with HEPES solution containing (in mM): 125 NaCl, 5 KCl, 1.2 MgSO_4_, 2 Na_2_HPO_4_, 32 HEPES, 5 glucose, 1 CaCl_2_, pH 7.4. Ca^2+^-free conditions were achieved by using Ca^2+^-free HEPES solution containing (in mM): 125 NaCl, 5 KCl, 1.2 MgSO_4_, 2 Na_2_HPO_4_, 32 HEPES, 5 glucose, 0.5 EGTA, pH 7.4.

### 4.5. Statistical Analysis

Statistical analysis was conducted using SPSS software (Version 25.0, SPSS Inc., Chicago, IL, USA). Data are provided as means ± SEM, and *n* represents the number of independent experiments (i.e., in fluorescence experiments, the number of dishes measured). All data were tested for significance using Student’s *t* test or ANOVA. Results with *p* < 0.05 were considered statistically significant.

## Figures and Tables

**Table 1:** List of the primer sequences for RT-PCR.

Name	Orientation	Sequence
*GAPDH*	Forward	5′-TCAAGGCTGAGAACGGGAAG-3′
	Reverse	5’-TGGACTCCACGACGTACTCA-3′
*CaSR*	Forward	5’-ATGCCAAGAAGGGAGAAAGACTCTT-3′
	Reverse	5′-TCAGGACACTCCACACACTCAAAG-3′
*NFAT5*	Forward	5′-GGGTCAAACGACGAGATTGTG-3′
	Reverse	5′-GTCCGTGGTAAGCTGAGAAAG-3′
*SGK1*	Forward	5′-AGGAGGATGGGTCTGAACGA-3′
	Reverse	5′-GGGCCAAGGTTGATTTGCTG-3′
*ORAI1*	Forward	5′-CACCTGTTTGCGCTCATGAT-3′
	Reverse	5′-GGGACTCCTTGACCGAGTTG-3′
*ORAI2*	Forward	5′-CAGCTCCGGGAAGGAACGTC-3′
	Reverse	5′-CTCCATCCCATCTCCTTGCG-3′
*ORAI3*	Forward	5′-CTTCCAATCTCCCACGGTCC-3′
	Reverse	5′-GTTCCTGCTTGTAGCGGTCT-3′
*STIM1*	Forward	5′-AAGAAGGCATTACTGGCGCT-3′
	Reverse	5′-GATGGTGTGTCTGGGTCTGG-3′
*STIM2*	Forward	5′-AGGGGATTCGCCTGTAACTG-3′
	Reverse	5′-GGTTTACTGTCGTTGCCAGC-3′

## Data Availability

The data presented in this study are available on request from the corresponding author.

## Supplementary Materials

Supplementary materials can be found at https://www.mdpi.com/1422-0067/22/7/3292/s1.

## References

[1] Kapustin A.N., Chatrou M.L., Drozdov I., Zheng Y., Davidson S.M., Soong D., Furmanik M., Sanchis P., De Rosales R.T., Alvarez-Hernandez D. (2015). Vascular smooth muscle cell calcification is mediated by regulated exosome secretion. Circ. Res..

[2] Lang F., Ritz E., Alesutan I., Voelkl J. (2014). Impact of aldosterone on osteoinductive signaling and vascular calcification. Nephron. Physiol..

[3] Lang F., Ritz E., Voelkl J., Alesutan I. (2013). Vascular calcification--is aldosterone a culprit?. Nephrol. Dial. Transpl..

[4] Steitz S.A., Speer M.Y., Curinga G., Yang H.Y., Haynes P., Aebersold R., Schinke T., Karsenty G., Giachelli C.M. (2001). Smooth muscle cell phenotypic transition associated with calcification: Upregulation of Cbfa1 and downregulation of smooth muscle lineage markers. Circ Res..

[5] Blacher J., Guerin A.P., Pannier B., Marchais S.J., London G.M. (2001). Arterial calcifications, arterial stiffness, and cardiovascular risk in end-stage renal disease. Hypertension.

[6] London G.M., Guerin A.P., Marchais S.J., Metivier F., Pannier B., Adda H. (2003). Arterial media calcification in end-stage renal disease: Impact on all-cause and cardiovascular mortality. Nephrol. Dial. Transpl..

[7] Mizobuchi M., Towler D., Slatopolsky E. (2009). Vascular calcification: The killer of patients with chronic kidney disease. J. Am. Soc. Nephrol..

[8] Foley R.N., Parfrey P.S., Sarnak M.J. (1998). Epidemiology of cardiovascular disease in chronic renal disease. J. Am. Soc. Nephrol..

[9] Alesutan I., Musculus K., Castor T., Alzoubi K., Voelkl J., Lang F. (2015). Inhibition of Phosphate-Induced Vascular Smooth Muscle Cell Osteo-/Chondrogenic Signaling and Calcification by Bafilomycin A1 and Methylamine. Kidney Blood Press Res..

[10] Feger M., Alesutan I., Castor T., Mia S., Musculus K., Voelkl J., Lang F. (2015). Inhibitory effect of NH4Cl treatment on renal Tgfss1 signaling following unilateral ureteral obstruction. Cell Physiol. Biochem..

[11] Lang F., Guelinckx I., Lemetais G., Melander O. (2017). Two Liters a Day Keep the Doctor Away? Considerations on the Pathophysiology of Suboptimal Fluid Intake in the Common Population. Kidney Blood Press Res..

[12] Leibrock C.B., Alesutan I., Voelkl J., Pakladok T., Michael D., Schleicher E., Kamyabi-Moghaddam Z., Quintanilla-Martinez L., Kuro-o M., Lang F. (2015). NH_4_Cl Treatment Prevents Tissue Calcification in Klotho Deficiency. J. Am. Soc. Nephrol..

[13] Chen S., Grigsby C.L., Law C.S., Ni X., Nekrep N., Olsen K., Humphreys M.H., Gardner D.G. (2009). Tonicity-dependent induction of Sgk1 expression has a potential role in dehydration-induced natriuresis in rodents. J. Clin. Investig..

[14] Lang F., Stournaras C., Zacharopoulou N., Voelkl J., Alesutan I. (2018). Serum- and glucocorticoid-inducible kinase 1 and the response to cell stress. Cell Stress.

[15] Lang F., Shumilina E. (2013). Regulation of ion channels by the serum- and glucocorticoid-inducible kinase SGK1. FASEB J..

[16] Ma K., Liu P., Al-Maghout T., Sukkar B., Cao H., Voelkl J., Alesutan I., Pieske B., Lang F. (2019). Phosphate-induced ORAI1 expression and store-operated Ca(2+) entry in aortic smooth muscle cells. J. Mol. Med..

[17] Zhu X., Ma K., Zhou K., Voelkl J., Alesutan I., Leibrock C., Nurnberg B., Lang F. (2020). Reversal of phosphate-induced ORAI1 expression, store-operated Ca(2+) entry and osteogenic signaling by MgCl_2_ in human aortic smooth muscle cells. Biochem. Biophys. Res. Commun..

[18] Ma K., Sukkar B., Zhu X., Zhou K., Cao H., Voelkl J., Alesutan I., Nurnberg B., Lang F. (2020). Stimulation of ORAI1 expression, store-operated Ca(2+) entry, and osteogenic signaling by high glucose exposure of human aortic smooth muscle cells. Pflug. Arch. Eur. J. Physiol..

[19] Lee S.H., Park Y., Song M., Srikanth S., Kim S., Kang M.K., Gwack Y., Park N.H., Kim R.H., Shin K.H. (2016). Orai1 mediates osteogenic differentiation via BMP signaling pathway in bone marrow mesenchymal stem cells. Biochem. Biophys. Res. Commun..

[20] Eckstein M., Vaeth M., Aulestia F.J., Costiniti V., Kassam S.N., Bromage T.G., Pedersen P., Issekutz T., Idaghdour Y., Moursi A.M. (2019). Differential regulation of Ca(2+) influx by ORAI channels mediates enamel mineralization. Sci. Signal..

[21] McCarl C.A., Picard C., Khalil S., Kawasaki T., Rother J., Papolos A., Kutok J., Hivroz C., Ledeist F., Plogmann K. (2009). ORAI1 deficiency and lack of store-operated Ca^2+^ entry cause immunodeficiency, myopathy, and ectodermal dysplasia. J. Allergy Clin. Immunol..

[22] Braun A., Varga-Szabo D., Kleinschnitz C., Pleines I., Bender M., Austinat M., Bosl M., Stoll G., Nieswandt B. (2009). Orai1 (CRACM1) is the platelet SOC channel and essential for pathological thrombus formation. Blood.

[23] Lang F., Munzer P., Gawaz M., Borst O. (2013). Regulation of STIM1/Orai1-dependent Ca^2+^ signalling in platelets. Thromb. Haemost..

[24] Eylenstein A., Gehring E.M., Heise N., Shumilina E., Schmidt S., Szteyn K., Munzer P., Nurbaeva M.K., Eichenmuller M., Tyan L. (2011). Stimulation of Ca^2+^-channel Orai1/STIM1 by serum- and glucocorticoid-inducible kinase 1 (SGK1). FASEB J..

[25] Borst O., Schmidt E.M., Munzer P., Schonberger T., Towhid S.T., Elvers M., Leibrock C., Schmid E., Eylenstein A., Kuhl D. (2012). The serum- and glucocorticoid-inducible kinase 1 (SGK1) influences platelet calcium signaling and function by regulation of Orai1 expression in megakaryocytes. Blood.

[26] Lang F., Gawaz M., Borst O. (2015). The serum- & glucocorticoid-inducible kinase in the regulation of platelet function. Acta Physiol..

[27] Sahu I., Pelzl L., Sukkar B., Fakhri H., Al-Maghout T., Cao H., Hauser S., Gutti R., Gawaz M., Lang F. (2017). NFAT5-sensitive Orai1 expression and store-operated Ca(2+) entry in megakaryocytes. FASEB J..

[28] Balduini A., Badalucco S., Pugliano M.T., Baev D., De Silvestri A., Cattaneo M., Rosti V., Barosi G. (2011). In vitro megakaryocyte differentiation and proplatelet formation in Ph-negative classical myeloproliferative neoplasms: Distinct patterns in the different clinical phenotypes. PLoS ONE.

[29] Golfier S., Kondo S., Schulze T., Takeuchi T., Vassileva G., Achtman A.H., Graler M.H., Abbondanzo S.J., Wiekowski M., Kremmer E. (2010). Shaping of terminal megakaryocyte differentiation and proplatelet development by sphingosine-1-phosphate receptor S1P4. FASEB J..

[30] Pelzl L., Sahu I., Ma K., Heinzmann D., Bhuyan A.A.M., Al-Maghout T., Sukkar B., Sharma Y., Marini I., Rigoni F. (2020). Beta-Glycerophosphate-Induced ORAI1 Expression and Store Operated Ca(2+) Entry in Megakaryocytes. Sci. Rep..

[31] Di Buduo C.A., Moccia F., Battiston M., De Marco L., Mazzucato M., Moratti R., Tanzi F., Balduini A. (2014). The importance of calcium in the regulation of megakaryocyte function. Haematologica.

[32] Somasundaram B., Mahaut-Smith M.P. (1994). Three cation influx currents activated by purinergic receptor stimulation in rat megakaryocytes. J. Physiol..

[33] Somasundaram B., Mason M.J., Mahaut-Smith M.P. (1997). Thrombin-dependent calcium signalling in single human erythroleukaemia cells. J. Physiol..

[34] Tolhurst G., Carter R.N., Amisten S., Holdich J.P., Erlinge D., Mahaut-Smith M.P. (2008). Expression profiling and electrophysiological studies suggest a major role for Orai1 in the store-operated Ca^2+^ influx pathway of platelets and megakaryocytes. Platelets.

[35] Alesutan I., Tuffaha R., Auer T., Feger M., Pieske B., Lang F., Voelkl J. (2017). Inhibition of osteo/chondrogenic transformation of vascular smooth muscle cells by MgCl_2_ via calcium-sensing receptor. J. Hypertens..

[36] House M.G., Kohlmeier L., Chattopadhyay N., Kifor O., Yamaguchi T., Leboff M.S., Glowacki J., Brown E.M. (1997). Expression of an extracellular calcium-sensing receptor in human and mouse bone marrow cells. J. Bone Miner. Res. Off. J. Am. Soc. Bone Miner. Res..

[37] Wang Y., Zhao Z., Shi S., Gao F., Wu J., Dong S., Zhang W., Liu Y., Zhong X. (2017). Calcium sensing receptor initiating cystathionine-gamma-lyase/hydrogen sulfide pathway to inhibit platelet activation in hyperhomocysteinemia rat. Exp. Cell Res..

[38] Zhang W., Sun R., Zhong H., Tang N., Liu Y., Zhao Y., Zhang T., He F. (2019). CaSR participates in the regulation of vascular tension in the mesentery of hypertensive rats via the PLCIP3/ACV/cAMP/RAS pathway. Mol. Med. Rep..

[39] Guo S., Yan T., Shi L., Liu A., Zhang T., Xu Y., Jiang W., Yang Q., Yang L., Liu L. (2021). Matrine, as a CaSR agonist promotes intestinal GLP-1 secretion and improves insulin resistance in diabetes mellitus. Phytomedicine.

[40] Maltsev A.V. (2018). Agmatine modulates calcium handling in cardiomyocytes of hibernating ground squirrels through calcium-sensing receptor signaling. Cell Signal..

[41] Ortiz-Capisano M.C., Reddy M., Mendez M., Garvin J.L., Beierwaltes W.H. (2013). Juxtaglomerular cell CaSR stimulation decreases renin release via activation of the PLC/IP(3) pathway and the ryanodine receptor. Am. J. Physiol. Ren. Physiol..

[42] Kwak J.O., Kwak J., Kim H.W., Oh K.J., Kim Y.T., Jung S.M., Cha S.H. (2005). The extracellular calcium sensing receptor is expressed in mouse mesangial cells and modulates cell proliferation. Exp. Mol. Med..

[43] Hattori T., Ara T., Fujinami Y. (2011). Pharmacological evidences for the stimulation of calcium-sensing receptors by nifedipine in gingival fibroblasts. J. Pharm. Pharm..

[44] Berna-Erro A., Jardin I., Smani T., Rosado J.A. (2016). Regulation of Platelet Function by Orai, STIM and TRP. Adv. Exp. Med. Biol..

[45] Renga B., Scavizzi F. (2017). Platelets and cardiovascular risk. Acta Cardiol.

[46] Moody W.E., Edwards N.C., Chue C.D., Ferro C.J., Townend J.N. (2013). Arterial disease in chronic kidney disease. Heart.

[47] Webster A.C., Nagler E.V., Morton R.L., Masson P. (2017). Chronic Kidney Disease. Lancet.

[48] Giachelli C.M., Jono S., Shioi A., Nishizawa Y., Mori K., Morii H. (2001). Vascular calcification and inorganic phosphate. Am. J. Kidney Dis..

[49] Shioi A., Nishizawa Y., Jono S., Koyama H., Hosoi M., Morii H. (1995). Beta-glycerophosphate accelerates calcification in cultured bovine vascular smooth muscle cells. Arter. Thromb. Vasc. Biol..

[50] Moe S.M., Chen N.X. (2004). Pathophysiology of vascular calcification in chronic kidney disease. Circ. Res..

[51] Abdelazeem K.N.M., Droppova B., Sukkar B., Al-Maghout T., Pelzl L., Zacharopoulou N., Ali Hassan N.H., Abdel-Fattah K.I., Stournaras C., Lang F. (2019). Upregulation of Orai1 and STIM1 expression as well as store-operated Ca(2+) entry in ovary carcinoma cells by placental growth factor. Biochem. Biophys. Res. Commun..

[52] Zhang S., al-Maghout T., Bissinger R., Zeng N., Pelzl L., Salker M.S., Cheng A., Singh Y., Lang F. (2017). Epigallocatechin-3-gallate (EGCG) up-regulates miR-15b expression thus attenuating store operated calcium entry (SOCE) into murine CD4+ T cells and human leukaemic T cell lymphoblasts. Oncotarget.

[53] Pelzl L., Hauser S., Elsir B., Sukkar B., Sahu I., Singh Y., Hoflinger P., Bissinger R., Jemaa M., Stournaras C. (2017). Lithium Sensitive ORAI1 Expression, Store Operated Ca(2+) Entry and Suicidal Death of Neurons in Chorea-Acanthocytosis. Sci. Rep..

[54] Sukkar B., Hauser S., Pelzl L., Hosseinzadeh Z., Sahu I., Al-Maghout T., Bhuyan A.A.M., Zacharopoulou N., Stournaras C., Schols L. (2018). Inhibition of Lithium Sensitive Orai1/STIM1 Expression and Store Operated Ca^2+^ Entry in Chorea-Acanthocytosis Neurons by NF-kappaB Inhibitor Wogonin. Cell Physiol. Biochem..

[55] Schmid E., Bhandaru M., Nurbaeva M.K., Yang W., Szteyn K., Russo A., Leibrock C., Tyan L., Pearce D., Shumilina E. (2012). SGK3 regulates Ca(2+) entry and migration of dendritic cells. Cell Physiol. Biochem..

