# Supplementary material for: Effect of MgCl2 and GdCl3 on ORAI1 Expression and Store-Operated Ca2+ Entry in Megakaryocytes
Source: Int J Mol Sci. 2021 Mar 24;22(7):3292. doi: 10.3390/ijms22073292 (PMC8036595; doi:10.3390/ijms22073292)
Supplement: Supplementary file 1 [file ijms-22-03292-s001.pdf]

Supplementary materials

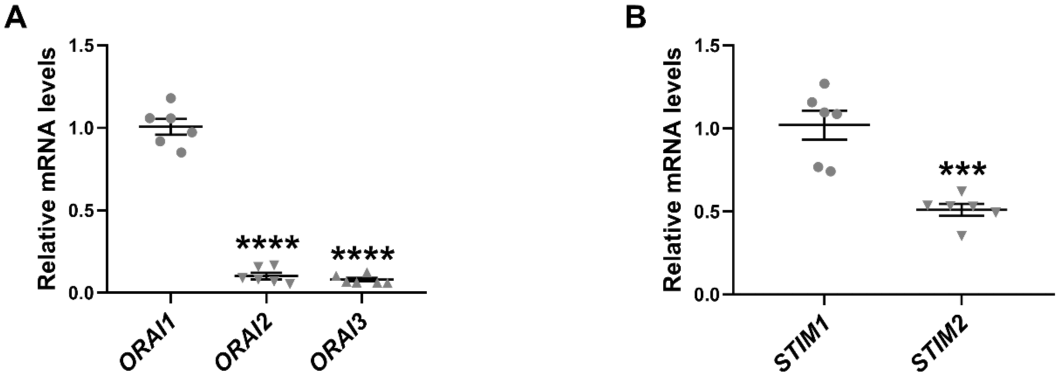

Figure S1. Relative transcription levels of *ORAI* and *STIM* isoforms in megakaryocytes. **A, B**. Arithmetic means (± SEM, $n = 6$) of *ORAI* **(A)** and *STIM* **(B)** isoforms mRNA expression under control conditions in megakaryocytes. The relative expression of *ORAI2* and *ORAI3* were normalized to *ORAI1* expression, while *STIM2* relative expression was normalized to *STIM1*. ***($p < 0.001$), ****($p < 0.0001$) indicates statistically significant difference to *ORAI1* or *STIM1* (ANOVA or Student's t test).
